# Dynamic thermo-mechanical properties of various flowable resin composites

**DOI:** 10.4317/jced.53061

**Published:** 2016-12-01

**Authors:** Stéphanie Jager, Rémy Balthazard, Marin Vincent, Abdessellam Dahoun, Eric Mortier

**Affiliations:** 1 DDS, research fellow, Département d’Odontologie Pédiatrique, Faculté d’Odontologie, 54000 Nancy, France; 2 DDS, PhD, Département d’Odontologie Conservatrice, Faculté d’Odontologie, 54000 Nancy, France; 3 DDS, research fellow, Département d’Odontologie Conservatrice, Faculté d’Odontologie, 54000 Nancy, France; 4 Professor, Institut Jean Lamour UMR 7198 CNRS, Université de Lorraine, Parc de Saurupt, 54011 Nancy Cedex, France; 5 DDS, PhD, Institut Jean Lamour UMR 7198 CNRS, Université de Lorraine, Parc de Saurupt, 54011 Nancy Cedex, France

## Abstract

**Background:**

This study compared the storage modulus (E’), the loss modulus (E’’) and the loss tangent (tan δ) of various flowable resin composites.

**Material and Methods:**

Grandio Flow (GRF), GrandioSo Heavy Flow (GHF), Filtek Supreme XTE (XTE) and Filtek Bulk Fill (BUL) flowable resins and Clinpro Sealant (CLI) ultra-flowable pit and fissure sealant resin were used. 25 samples were tested using a dynamical mechanical thermal analysis system in bending mode. Measurements were taken within a temperature range of 10 to 55°C. The results were statistically analyzed using mixed-effect and repeated-measure analysis of variance followed by paired multiple comparisons.

**Results:**

For all the materials, the E’ values decrease with temperature, whereas the tan δ values increase. Irrespective of the temperature, GHF and GRF present E’ and E’’ values significantly higher than all the other materials and CLI presents values significantly lower than all the other materials. Observation of the values for all the materials reveals a linear progression of the tan δ values with temperature.

**Conclusions:**

A variation in temperature within a physiological range generates modifications in mechanical properties without damaging the material, however. Filler content in volume terms appears to be the crucial parameter in the mechanical behavior of tested materials.

** Key words:**Dynamic mechanical thermal analysis, elastic modulus, filler content, flowable resin composites, loss modulus, loss tangent.

## Introduction

Adhesive and preventive dentistry, ultraconservative dentistry and esthetic dentistry have all developed significantly in recent decades and are now an integral part of the treatment arsenal available to practitioners. In the past fifteen or so years, the emergence of flowable resin composites has further expanded the range of options available to practitioners. The relative ease of use of these low-viscosity resins, their capacity to spread and take shape in small occlusal or cervical cavities, as well as their ability to penetrate into pits and fissures mean that they have been adopted by numerous dental practitioners. Resin composites are heterogeneous materials primarily made up of three components: matrix resin based on dimethacrylate monomers, mineral or organo-mineral fillers and a coupling agent, such as silane, to link the matrix and fillers chemically and prevent these fillers from acting as stress concentrators. To make resin composite flowable, manufacturers can proceed in two ways. The first option consists in increasing the proportion of viscosity-lowering monomers (so-called diluents) in order to counter the high viscosity of high-molecular weight monomers such as bisphenol A glycidic dimethacrylate (Bis-GMA) and, to a lesser extent, urethane dimethacrylate (UDMA). These are small low-molecular weight aliphatic monomers; triethylene glycol dimethacrylate (TEGDMA) is very frequently used for this purpose, for example. The second option consists in reducing the filler content in terms of volume compared to the matrix volume. Obviously, the two options can be employed simultaneously to varying degrees. Consequently, these specific composition characteristics will have an influence on the properties of these resin composites.

Resin composites can be considered to be viscoelastic materials (1,2). A purely elastic material is capable of storing all the energy applied to it during deformation in order to regain its initial shape when the stress ceases. Conversely, a purely viscous material loses all the energy applied to it during deformation. As for a viscoelastic material, when it is deformed, it stores a proportion of the energy applied while another proportion is dissipated in the form of heat. When the stress ceases, the viscoelastic material will partially regain its initial shape via its elastic portion and partially be permanently deformed due to its viscous portion. If only static methods are used to assess the mechanical properties of resin composites, only the elastic portion will be considered. In contrast, the use of dynamic tests appears to be particularly useful since these assess both the elastic response and the viscous response of the material (3). Furthermore, dynamic tests better mimic the physiological cyclical masticatory loading to which the materials are subjected in clinical use (4). Consequently, dynamic mechanical thermal analysis (DMTA) appears to be ideal for predictive assessment of the clinical behavior of materials (3,5).

No study of dynamic thermo-mechanical properties of flowable resin composites was found in the literature so far.

The aim of this work is to use DMTA to study the storage modulus (E’), the loss modulus (E’’) and the loss tangent (tan δ) of four flowable resin composites and one ultra-flowable pit and fissure sealant resin used in conservative dentistry, as a function of temperature.

## Material and Methods

 Table 1 lists the five materials used in the study. They are four flowable resin composites (Grandio™ Flow and GrandioSo™ Heavy Flow, Voco, Cuxhaven, Germany; Filtek™ Supreme XTE and Filtek™ Bulk Fill, 3M Espe, St Paul, MN, USA) and one ultra-flowable pit and fissure sealant resin (Clinpro™ Sealant, 3M Espe, St Paul, MN, USA). The decision to include an ultra-flowable pit and fissure sealant resin in the materials studied is guided by a desire to record the potential impact of extremely low filler content.

**Table 1:**
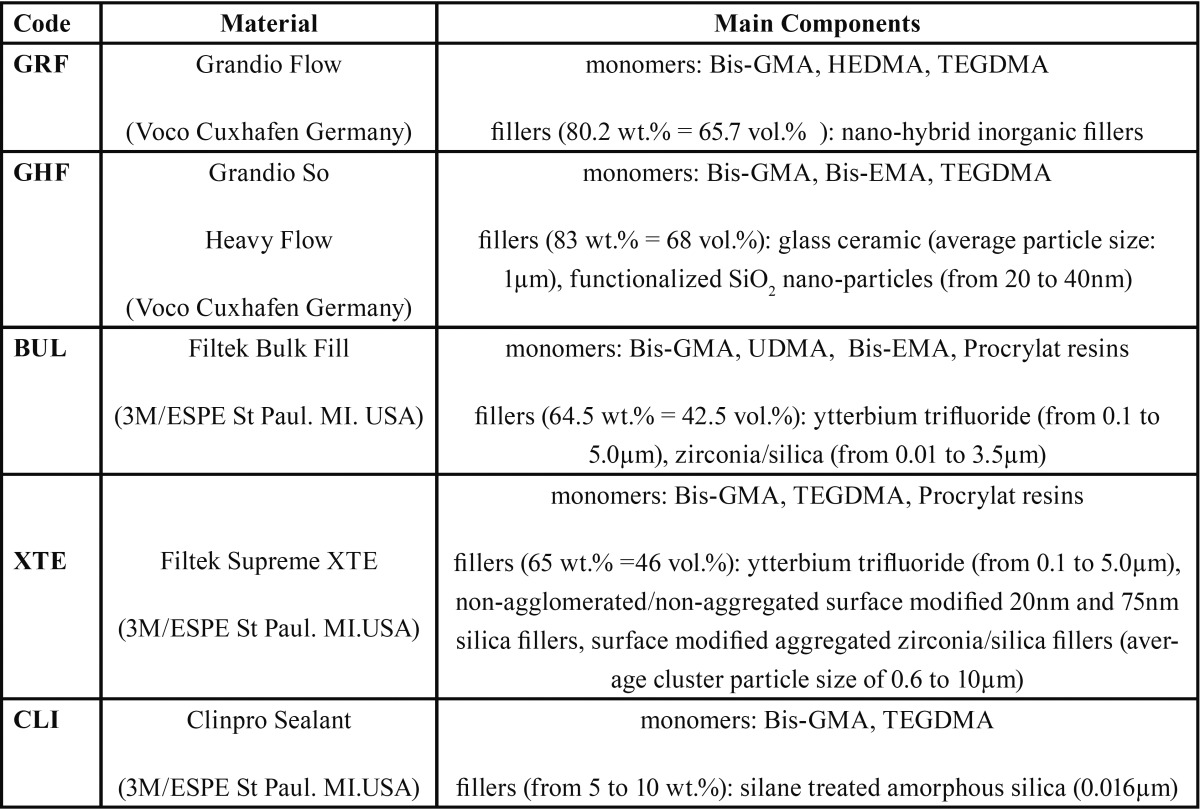
Materials used in this study.

25 bar-shaped specimens were prepared. The samples were prepared using a Teflon mold (2x2x25mm). For each sample, the material was injected into the mold using the applicator nozzles supplied by the manufacturers, remaining in contact with one wall of the mold to minimize air inclusion. A glass slide with a thickness of 1.10 mm was then firmly applied and held on the mold using a clamp to compress the material and eliminate any excess. Polymerization was performed using an LED lamp (Elipar Freelight 2, 3M Espe, St Paul, MN, USA) at three different points through the glass slide. Each light exposure lasted 40 s. The samples were then carefully removed from the molds. The side opposite the one already photo-polymerized was also exposed at 3 different points for 40 s per light exposure. Each sample was thus photo-polymerized for a total of 240 s. Excess material was carefully removed using a scalpel blade then the samples were gently polished by hand using abrasive paper with a particle size of 15 µm (P1200 ISO 6344). The exact dimensions of each of the samples were then measured and recorded using an electronic slide gauge (Digimatic, model 500-181U, Mitutoyo Corporation, Tokyo, Japan). Samples were immediately tested. The mechanical tests were performed using a dynamic mechanical analysis system (DMA 242C, Netzsch Gerätebau GmbH, Selb, Germany) in bending mode at a stress frequency of 1 Hz with oscillation amplitude of 20 µm and an applied force of 5 N. The measurements were taken within a temperature range of 10 to 55°C, with an increase of 2°C per minute. The E’ and tan δ values were recorded throughout this period. The E’’ values were then calculated using the formula E’’= E’ x tan δ.

The results were statistically analyzed using mixed-effect and repeated-measure analysis of variance followed by paired multiple comparisons (Bonferroni adjustment).

## Results

**Figure 1:**
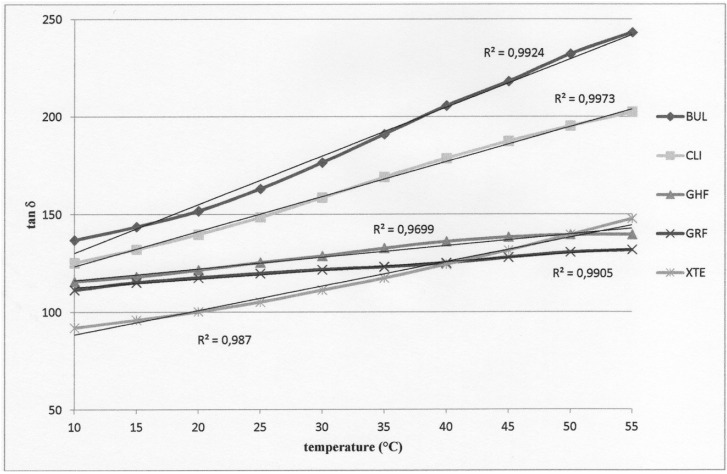
Loss tangent (tan δ) as a function of temperature (°C) (each data point is an average of five measurements); trend curves and determination coefficient R2 resulting from tan δ values.

The results are presented in  Table 2 to  Table 4 and in figure 1.

**Table 2:**
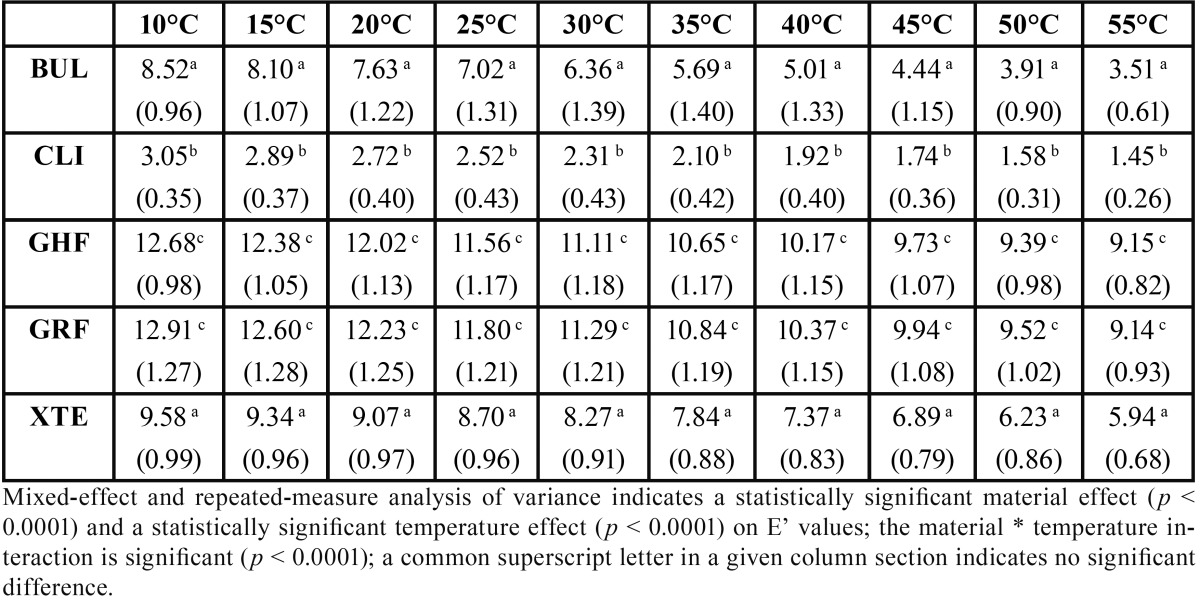
Mean values (SD) of storage modulus as a function of temperature (°C) (n=5).

**Table 3:**
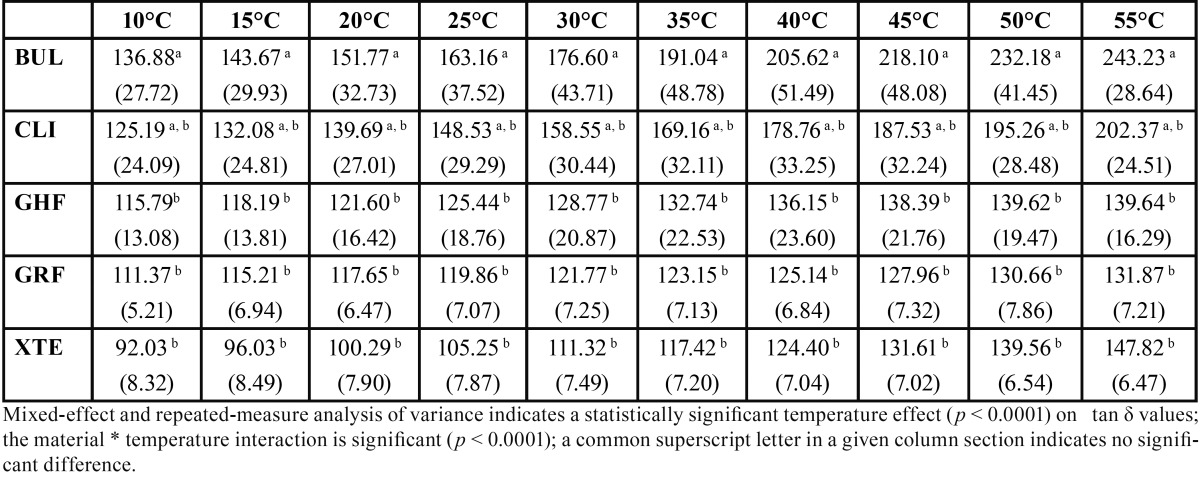
Mean values (SD) of loss tangent (tan δ) as a function of temperature (°C) (n=5).

**Table 4:**
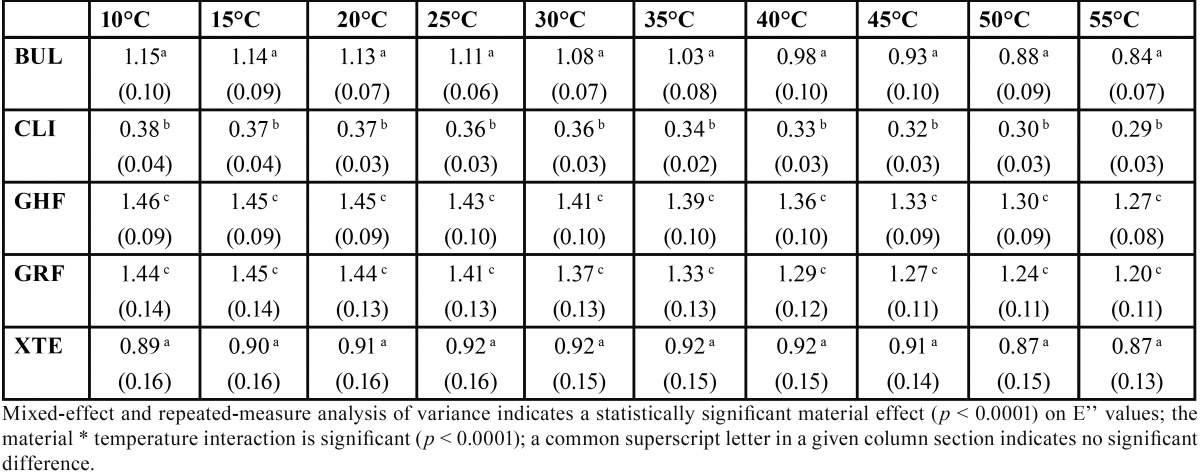
Mean values (SD) of loss modulus E’’ (GPa) as a function of temperature (°C) (n=5).

Mixed-effect and repeated-measure analysis of variance indicates a statistically significant material effect (*p* < 0.0001) on E’ and E’’ values and a statistically significant temperature effect (*p* < 0.0001) on E’ and tan δ values. The material * temperature interaction is significant (*p* < 0.0001), indicating an effect of temperature on the E’, tan δ and E’’ values for each of the materials.

The Bonferroni adjusted multiple comparison tests indicate, irrespective of the temperature, significantly higher E’ and E’’ values, but without any difference for GHF and GRF compared to all the other materials and significantly lower E’ and E’’ values for CLI compared to all the other materials. The E’ and E’’ values are not significantly different for XTE and BUL. The Bonferroni adjusted multiple comparison tests indicate, irrespective of the temperature, significantly higher tan δ values for BUL compared to GHF, GRF and XTE and with no difference with CLI, while CLI, XTE, GRF and GHF do not present tan δ values significantly different from one another. Observation of the values for all the materials reveals a linear progression of the tan δ values with temperature.

## Discussion

The resin composites used in dentistry are heterogeneous materials presenting viscoelastic behavior due to the resin matrix used in their composition. Consequently, dynamic analyses appear to be more appropriate than static methods to assess their mechanical capacity to meet the specifications for which they have been designed. In a DMTA measurement, the sample is subjected to a sinusoidal dynamic stress at a given frequency. The sample responds to this stress with a sinusoidal dynamic deformation of the same frequency as the force, but with a certain phase shift. In bending mode, the storage modulus E’, which characterizes the elastic behavior, and the loss modulus E’’, which measures the mechanical energy dissipated in the form of heat during elastic deformation and represents the viscous nature of the material, can be determined. The ratio between the energy dissipated by damping and the elastic energy stored then restored during a sinusoidal deformation cycle can be obtained. This is known as the loss tangent or the tangent δ damping coefficient, which is equal to E’’/E’.

In our study, it was decided to include an ultra-flowable pit and fissure sealant resin, in full awareness of its narrower scope of application to that of flowable restorative composites, given that the latter are also indicated for sealing pits and fissures, as well as for small fillings at occlusal and cervical sites, or to serve as a dentin substitute in sandwich fillings. However, in view of the very low filler content (5 to 10 wt.%, data not available in vol.%) of this pit and fissure sealant resin, it appears to be interesting to record the potential impact of such a characteristic leading to its very low viscosity in terms of mechanical properties.

In our study, at all the temperatures, the E’ values, representing the elastic component related to the stiffness of the material, are significantly higher for GHF and GRF compared to all the other materials, whereas they are significantly lower for CLI compared to all the other materials ( Table 2). This result is doubtless due to the proportion of fillers in volume terms in the different materials: at equivalent volume, the higher the filler proportion of a material, the lower its matrix volume, thereby explaining its greater stiffness. Braem proposed a mathematical method for determining the storage modulus based on the equation 

E= 3103.33e0.029771720X, in which X is the volumetric percentage of fillers and E is the dynamic elasticity modulus after storage in a dry place for 1 day (6). Giving sometimes over-estimated theoretical values, this formula clearly indicates the importance of the volumetric filler content (2). Here, GHF and GRF, with their respective contents of 68%vol. and 65.6%vol., do indeed have higher filler content than all the other materials. However, ideally, one might expect a restorative material to present the same structural, mechanical and physical characteristics of dentin (7). Yet the E’ modulus of dentin at 37°C and at 1 Hz was measured as being 15 GPa (8): in our study all the materials present values well below this at 35°C and 40°C, consistent with the results of other studies (7,9,10), indicating, in theory, that flowable resins are actually incapable of fully serving as dentin substitutes and the need to restrict them to small-volume restorations. Indeed, the greater the difference between the elasticity modulus of dentin and that of the material, the higher the risk of interface destruction appears to be (11,12). Intended exclusively as a pit and fissure sealant material, CLI is not concerned by these considerations, although its very low values tend to indicate the clinical requirement to position it away from any occlusal contact if its durability is to be guaranteed.

The significant influence of temperature on E’ values is also observed: it appears, in fact, that the modulus decreases as the temperature increases, as a result of the relaxation phenomena associated with the various degrees of freedom of the molecular chains. For our protocol, we deliberately chose to restrict ourselves to a temperature range of 10°C to 55°C, corresponding to a physiological range. For this temperature range, it is observed that the reduction in storage modulus is variable depending on the material, ranging from 30% to over 50%. Once again, the materials with the highest filler content - GHF and GRF - present a smaller reduction in E’ as temperature increases than the other materials.

During a dynamic measurement, a sinusoidal stress is applied at a determined frequency. The stress signal can then be written as σ(t) = σ0 . sin(ωt) where σ0 is the amplitude of the stress cycle, ω the pulse in rad/s and t the time. The response signal during deformation of a viscoelastic material will be out-of-phase because it dissipates a proportion of the energy by deforming itself. We then have ε(t) = ε0 . sin(ωt + δ) where ε0 is the amplitude of the deformation cycle. The phase shift between stress and deformation is then given by the phase angle δ. The tangent of the phase angle δ noted as tan δ is also known as the dissipation factor: this data indicates the vibration damping ability during mechanical deformation. Damping is a dimensionless property and is a measure of how well the material can disperse (absorb or emit) energy (13). In our study, materials BUL and CLI demonstrate a greater damping ability than GHF, GRF and XTE ( Table 3). Once again, a higher matrix volume proportion largely explains this result. However, with nonetheless very similar filler contents, BUL and XTE demonstrate different behaviors. The nature of the monomers included in their composition doubtless plays a major role in this difference. The polymer network of XTE resulting from Bis-GMA and TEGDMA with short inter-molecular distances is denser than that of BUL, which contains no TEGDMA. In addition, the probably more numerous hydrogen bonds in XTE, whereas these have been replaced in the Bis-EMA included in the composition of BUL, reduce the rotation capacity of the molecules, particularly at low temperatures (5). These two factors help explain the lower molecular mobility of XTE and hence its lower tan δ values than those of BUL. It can also be noted that for all the materials, tan δ values increase along with the temperature, this being accompanied with an increase in molecular mobility. This increase in tan δ values for GHF and GRF nonetheless occurs with a weaker slope than for the other materials, demonstrating a greater restriction in molecular movement despite the temperature increase (1). Generally speaking, if the isofrequency curve of tan δ is plotted as a function of temperature, the maximum tan δ value can be found, typically after a brief fall in the slope of the curve and just before its inflection. For this value, the temperature is known as the glass transition temperature Tg. At this temperature, the materials undergo marked variations in their physical properties. This temperature consists, to an extent, in the maximum use temperature of the material. In other words, at this glass transition temperature, the molecular chains will acquire an additional degree of freedom, they will be submitted to the mechanical stress imposed and the polymer will soften. In our study, we have deliberately restricted ourselves to a temperature range corresponding to the temperatures to which the materials may in reality be subjected inside the mouth. None of the materials present any inflection of tan δvalues between 10°C and 55°C, the values progress in a quasi-linear fashion without any modification in the slope (0.97 < R2 < 1.00 for all the materials) (Fig. 1), demonstrating that the glass transition temperature is not reached and that they are capable of tolerating the temperature increases that may occur in the mouth during the intake of food or liquid (13). The progression according to a weaker slope of the tan δ values for GHF and GRF appears to indicate, by extrapolation, a higher maximum use temperature, which is consistent with their lower potential molecular mobility (2). A low Tg value is also liable to indicate a polymerization deficiency, which is not the case here with, in our study protocol, 240s of light exposure per sample.

The loss modulus E’’ represents the viscous component of the material. A higher E’’ value reflects a greater capacity to dissipate mechanical energy in the form of heat during deformation. A high E’’ value is therefore a guarantee of the capacity to tolerate the strain during masticatory function stresses. In our study, at all temperatures, GHF and GRF present significantly higher E’’ values whereas CLI demonstrates significantly lower values ( Table 4). Once again, the filler content appears to be a prime factor. Indeed, it is the friction between the molecular chains and the filler particles that is potentially the greatest source of energy dissipation during deformation under stress. The lower capacity of CLI, with its very low filler content, to dissipate said energy can thus be easily understood. Conversely, this friction will be marked for resins with high filler content, such as GHF and GRF. That said, the type of filler, and the size and form of the particles, will therefore also play a role: these factors are probably the source of the difference in values obtained between XTE and BUL, which nonetheless present relatively similar filler contents in volume terms.

Flowable resin composites are now an integral part of the treatment arsenal available to practitioners. Their characteristics mean that they are frequently indicated in conservative dentistry. However, the term “flowable resin composites” covers numerous different products with diverse compositions and properties, which directly influence the scope of their indications. DMTA is an easy-to-use, precise method demonstrating excellent reproducibility for the mechanical characterization of viscoelastic materials, such as flowable resin composites. It appears, for the materials tested in our study using our experimental conditions, that a varia-tion in temperature within a physiological range of 10°C to 55°C generates modifications in mechanical properties without damaging the material, however. It also appears that the filler content in volume terms represents the crucial parameter in the mechani-cal behavior of said materials. Consequently, it should be clearly indicated on the instruction leaflets for flowable resin composites as one of the major criteria guiding practitioners’ choices.
